# Develop and Field Evolution of Single Tube Nested PCR, SYBRGreen PCR Methods, for the Diagnosis of Leprosy in Paraffin-embedded Formalin Fixed Tissues in Yunnan Province, a Hyper endemic Area of Leprosy in China

**DOI:** 10.1371/journal.pntd.0007731

**Published:** 2019-10-02

**Authors:** Xiaohua Chen, Yan Xing, Jun He, Fuyue Tan, Yuangang You, Yan Wen

**Affiliations:** 1 Beijing Tropical Medicine Research Institute, Beijing Friendship Hospital, Capital Medical University, Beijing, China; 2 Beijing Key Laboratory for Research on Prevention and Treatment of Tropical Diseases, Capital Medical University, Beijing, China; 3 The Centers for Disease Control and Prevention of Yunnan Province, Kunming, China; 4 Wenshan Institute of Dermatology, Wenshan Dermatology Hospital, The Alliance Hospital of The First Affiliated Hospital of Kunming Medical University, Yunnan, China; Hospital Infantil de Mexico Federico Gomez, UNITED STATES

## Abstract

**Background:**

Detection and pathology analysis of *Mycobacterium leprae* using skin biopsy tissues are essential for leprosy diagnosis and monitoring response to treatment. Although formalin fixation of patient tissues may not be ideal for molecular studies, biopsy samples are the most accessible material from suspected cases. Therefore, clinical molecular laboratories must be able to utilize formalin-fixed, paraffin-embedded (FFPE) material.

**Objective:**

To determine the best molecular method for diagnosing and monitoring leprosy in FFPE specimens, we developed a single-tube nested PCR (STNPCR) (131 bp) and SYBRGreen PCR (101 bp) assay using primers for the *M*. *leprae*-specific repetitive element (RLEP) gene and evaluated the results compared to those using previously established RLEP primers (372 bp).

**Methods:**

FFPE biopsy samples obtained from 145 leprosy patients (during or after multidrug therapy (MDT)) and patients with 29 other confounding dermatoses were examined by the bacteria index (BI) and by simple PCR, STNPCR, and SYBRGreen PCR using primers amplifying a 372-bp, 131-bp or 101-bp fragment of RLEP, respectively.

**Results:**

In leprosy patients receiving MDT, STNPCR showed a highest specificity of 100% and a positive predictive value (PPV) of 100%. For multibacillary (MB), paucibacillary (PB) and all leprosy patients, the highest sensitivities were 91.42%, 39.13%, and 67.92%, negative predictive values (NPVs) were 8.57%, 60.36%, and 32.07%, and the highest accuracies were 93.93%, 62.67%, and 74.81%, respectively, higher than the results of SYBRGreen PCR and simple PCR. For post-MDT leprosy patients, SYBRGreen PCR showed the highest sensitivity of 50.0%, highest specificity of 100%, a PPV of 100%, an NPV of 100% and the highest accuracy of 83.72% for MB patients, which were higher than those of STNPCR and simple PCR. STNPCR showed the highest sensitivity of 26.66% and 34.48%, highest specificity of 100% and 100%, a PPV of 100% and 100%, NPV of 72.50% and 60.21%, and highest accuracy of 75.00% and 67.24% for PB and leprosy patients, respectively, higher than those of SYBRGreen PCR and simple PCR.

**Conclusions:**

These findings suggest that STNPCR or SYBRGreen PCR (131-bp and 101-bp fragment amplification, respectively) for RLEP using FFPE specimens performs better as a diagnostic test and for monitoring response to MDT than does simple PCR based on 372-bp fragment amplification. Additionally, STNPCR showed increased sensitivity for PB diagnosis using FFPE specimens, which can be transferred remotely or retrieved from previous leprosy patients.

## Author summary

Leprosy is one of the oldest diseases known to humankind and is caused by *Mycobacterium leprae*. Despite being curable, leprosy is still a notorious disease, causing serious disability and a stigma generally associated with late diagnosis. The disease is challenging to diagnose because there is no gold standard method for detecting *M*. *leprae* or its cellular components (DNA, lipids or proteins). In past decades, different PCR methods were developed to amplify different *M*. *leprae* gene targets, such as the RLEP gene, with high sensitivity in leprosy diagnosis using skin biopsy and slit skin smear (SSS) specimens. However, few reports have focused on FFPE specimens. Because FFPE specimens can be transferred remotely or retrieved from previous leprosy patients, we developed and evaluated the STNPCR assay for detecting *M*. *leprae* in these specimens. Our results suggest that STNPCR of RLEP using FFPE specimens performs better as a diagnostic test and for monitoring response to MDT than does simple PCR.

## Introduction

Leprosy is one of the oldest diseases known to humankind and is caused by *Mycobacterium leprae*. Recently, due to scientific advances, knowledge has increased about the pathogenesis, prognosis and treatment of this stigmatized disease. Despite being curable, leprosy is still a notorious disease, causing serious disability and a stigma generally associated with late diagnosis. The implementation of World Health Organization (WHO) multidrug therapy (MDT) has drastically reduced the number of registered leprosy cases from approximately 12 million reported in 1985 to 211,973 new cases globally in 2015 (WHO, http://www.who.int/lep/epidemiology/en/) [1]. Nonetheless, the disease is challenging to diagnose because there is no gold standard method to detect *M*. *leprae* or its cellular components (DNA, lipids or proteins). Indeed, acid-fast bacilli (AFBs) in slit smears of the tuberculoid (TT), indeterminate and pure neural leprosy (PNL) forms are very rare or absent [2].

Over the past 20 years, PCR methods have been developed to amplify different *M*. *leprae* gene targets, including genes encoding various proteins, such as the 36-kDa antigen [3], the 18-kDa antigen [4], the 65-kDa antigen [5], Ag 85B [6], 16S rRNA [7] and the *M*. *leprae*-specific repetitive element (RLEP) [8]. Among most PCR-based studies, the 372-bp RLEP PCR approach is favored, as multiple copies are present in the genome [8].

Although formalin fixation and paraffin embedding of tissue may be suboptimal for molecular studies, such processing is firmly entrenched in pathology practice. Rather than seeking alternative fixatives, successful clinical molecular laboratories must be able to utilize formalin-fixed, paraffin-embedded (FFPE) material [9].

In the present study, we developed and evaluated the diagnostic and monitoring performance of single-tube nested PCR (STNPCR) and SYBRGreen PCR assays (based on amplification of 131-bp and 101-bp regions of the 372-bp RLEP fragment) and compared their performance with that of already-established simple PCR methods (based on amplification of the 372-bp RLEP fragment) used to diagnose leprosy using FFPE specimens collected from symptomatic patients visiting the Centers for Disease Control and Prevention (CDC) in the hyperendemic area of leprosy of Yunnan (YN) Province, southwestern China. Leprosy diagnosis criteria were used as a reference in addition to the PCR results.

## Methods

### Ethics statement

This study was approved by the Medical Ethics Committee of Beijing Friendship Hospital, Capital Medical University, Beijing, P. R. China. Written informed consent was obtained from all study participants or from their parents or guardians. Any patients diagnosed with leprosy by clinicians using defined criteria, slit skin smears and biopsies were treated with MDT appropriately according to WHO guidelines.

### Study design and population

The study was conducted in YN Province, mainly at the CDC of Wenshan Zhuang and Miao Autonomous Prefecture (WS), Honghe Hani and Yi Autonomous Prefecture (HH), and Kunming (KM) (the capital of YN). Subjects visiting the CDC between 2011 and 2013 were recruited for this study. Clinical, demographic, and immunohistopathological characteristics were recorded. In addition, 29 non-leprosy cases (with other confounding dermatoses) were enrolled as non-leprosy controls after informed consent was obtained for participation.

Pockets of endemicity in the ethnically diverse, mountainous and underdeveloped southwestern province of YN and its neighboring provinces Guizhou (GZ) and Sichuan (SC) in southwestern China account for the majority of leprosy cases in this country [10]. The number of new cases detected in YN was 1090 from 2005 to 2015 [11], and the detection rates in WS, HH and KM were 22.57% (246/1090), 16.79% (183/1090) and 8.35% (91/1090), respectively, for 2005–2015. These areas have the top three percentages of leprosy in YN.

### Study subject and samples

One hundred fifty leprosy patients were classified into five groups based on the Ridley and Jopling [12] classification: TT, borderline-tuberculoid (BT), borderline-borderline (BB), borderline-lepromatous (BL), and lepromatous (LL) groups. The leprosy patients were also classified into two groups for data analysis, PB and MB, according to the WHO operational classification [13] before or after MDT.

Twenty-nine patients with other confounding dermatoses (piebald, psoriasis, pityriasis rosea, body moss, multiple neurofibroma, multiplex lipoid granuloma, and scleroderma) from the same endemic region were included as non-leprosy controls.

For the study, FFPE samples (n = 174) were collected from symptomatic subjects attending the CDC. Current histology practice employs preservation of tissue by fixation in neutral buffered formalin prior to embedding in paraffin. Specimen processing involved cutting 10-mm-thick sections of paraffin-embedded tissue from each paraffin-embedded tissue block using a microtome blade; to prevent cross contamination, the microtome blade was cleaned with 100% ethanol after sectioning each sample, as described previously [14].

### DNA extraction

Ten slides of 10-mm-thick sections of paraffin-embedded formalin-fixed tissues were used to obtain genomic DNA for PCR. Genomic DNA (gDNA) was extracted at the reference laboratory in the Leprosy Department, Beijing Tropical Medicine Research Institute using DNeasy Blood and Tissue Kit (Qiagen, Inc., Valencia, CA) in accordance with the manufacturer’s instructions. The extracted DNA was quantified by measuring absorbance at 260 nm. A dilution series of standard DNA (0.05–1 ng) was used to evaluate the limit of detection of the systems. One microliter of isolated DNA was used as the template for each PCR system.

### Primer design

The available sequences for *M*. *leprae* RLEP from the National Center for Biotechnology Information (NCBI) database (http://www.ncbi.nlm.nih.gov/) were retrieved and aligned. The 372-bp fragment [15] was utilized to design primers for STNPCR (131 bp) and SYBRGreen PCR (101 bp) using Primer 5 software (PREMIER Biosoft., CA, USA).

### Simple PCR, STNPCR and SYBRGreen PCR

The simple PCR mixture included 250 nM of each primer (372 bp), 10 μl of 2X PCR Mixture (Cat No: MFKIT01, Beijing Jinsheng Lida Technology Trade Co., Ltd., Beijing, China), and 2–50 ng of DNA template (1 μl) in a 20-μl volume. Reactions were carried out using a Peltier Thermal Cycler (BIO-RAD, California, USA), and the thermal cycling conditions were as follows: 2 min at 94°C, followed by 35 cycles of 30 sec at 94°C, 30 sec at 60°C and 30 sec at 72°C, and a final extension step of 5 min at 72°C.

The STNPCR mixture with uracil N-glycosylase (UNG) contained 10 μl of 2×Taq PCR StarMix with Loading Dye with UNG, 16.7 nM or 250 nM of each primer set (372 bp or 131 bp, respectively) (cat no: MFKIT09, Beijing Jinsheng Lida Technology Trade Co., Ltd., Beijing, China), and 2–50 ng of DNA template (1 μl) in a 20-μl volume. The thermal cycling conditions were as follows: 5 min at 50°C, 15 min at 95°C, followed by 20 cycles of 30 sec at 94°C, 60 sec at 60°C and 30 sec at 72°C, 30 cycles of 30 sec at 94°C, 60 sec at 40°C and 30 sec at 72°C, and a final extension step of 10 min at 72°C. Products were visualized on a 1.5% agarose gel containing GeneFinder (Zeesan Biotech, Xiamen, Fujian, China).

SYBRGreen PCR was carried out to quantify levels of *M*. *leprae* in FFPE tissue samples using Applied Biosystems 7500 Real-Time PCR System (Applied Biosystems, Foster City, California, USA). For reactions using SYBRGreen chemistry, each 20-μl reaction consisted of 2–50 ng of genomic DNA (1 μl), 250 nM of each inner primer (see Table 1), 10 μl of 2X qPCR Mix (cat no: MFKIT08, Beijing Jinsheng Lida Technology Trade Co., Ltd., Beijing, China), and 8.2 μl of water. The thermal cycling conditions were as follows: 1 cycle of 95°C for 10 min, followed by 40 cycles of 95°C for 15 sec and 60°C for 1 min.

**Table 1:** Primers and probes used.

Target gene	Primers/probe	Nucleotide sequence (5'–3')	Product length	PCR type	Ref
RLEP	Forward primer	5-CGGCCGGATCATCGATGCAC-3′	372 bp	Simple PCR/STNPCR (outer)	[15]
	Reverse primer	5-GCACGTAAGCATGTCGGTGG-3′			
	Forward primer	5-GTGAGGGTAGTTGTT-3′	131 bp	STNPCR (inner)	This study
	Reverse primer	5-GGTGCGAATAGTT-3′			
	Forward primer	5'-GTGCATGTCATGGCCTTGAG-3'	101 bp	SYBRGreen PCR	This study
	Reverse primer	5'-GGGATAACATCAGGTGCGAATAGTT-3'			

RLEP = M. leprae-specific repetitive element; bp = base pair; PCR = polymerase chain reaction; STNPCR = single-tube nested PCR.

### Statistical analysis

Data were entered into VassarStats: Website for Statistical Computation (http://vassarstats.net/index.html). The sensitivity, specificity, positive predictive value (PPV), negative predictive value (NPV), positive likelihood ratio (LR+) and negative likelihood ratio (LR-) with 95% confidence intervals (CIs) were calculated using calculator 1 of VassarStats: Website for Statistical Computation. The calculation for accuracy was as follows: accuracy = [true positives + true negatives]/total specimens.

## Results

### Developed primers for STNPCR and SYBRGreen PCR

A comparison of the primer sequences with those in the nucleotide collection database of NCBI showed no cross-reactivity between the primer set and common human pathogens and the ability to amplify the respective targets. For STNPCR, we chose to use concentrations of outer and inner primers of 20 nM and 250 nM and annealing temperatures of 60°C and 40°C in the first and second stages, respectively. The sequences and details for the primers are listed in Table 1.

### Analytical sensitivity of PCR methods

Analytical sensitivity represents the smallest amount of template in the sample that can be accurately detected by an assay. The detection limit of RLEP by simple PCR (372 bp), STNPCR (131 bp) and SYBRGreen PCR (101 bp) was the same, at 13 fg DNA.

### Analytical specificity of PCR methods

The specificity of the primers was tested by carrying out amplification with purified genomic DNA from fifteen different mycobacterial species and four non-mycobacterial species. All primer sets showed amplicons when DNA isolated from *M*. *lepraemurium* was used as the template. Conversely, no primer sets amplified any region when DNA from other species of *Mycobacterium* or non-mycobacterial species was used as the template (see Table 2).

**Table 2:** Specificity of RLEP by simple PCR, STNPCR, and SYBRGreen PCR for *Mycobacterium leprae* detection.

Species	RLEP		
	Simple PCR	STNPCR	SYBRGreen PCR
**Mycobacterial species**	from CSU* and NHDP**		
*M*. *lepraemurium*	+	+	+
*M*. *avium*	-	-	-
*M*. *bovis BCG-Pasteur*	-	-	-
*M*. *bovis [AFZ/ZZ/97]*	-	-	-
*M*. *bovis[Ravenel]*	-	-	-
*M*. *chelonae*	-	-	-
*M*. *flavescens*	-	-	-
*M*. *gordonae*	-	-	-
*M*. *intracellulare*	-	-	-
*M*. *kansasii*	-	-	-
*M*. *marinum*	-	-	-
*M*. *phlei*	-	-	-
*M*. *smegmatis*	-	-	-
*M*. *simiae*	-	-	-
*M*. *ulcerans*	-	-	-
**Non-mycobacterial species**	from NHDP**		
*Streptococcus pyogenes*	-	-	-
*Clostridium perfringens*	-	-	-
*Escherichia coli*	-	-	-
*Staphylococcus epidermidis*	-	-	-

*CSU = Colorado State University, Fort Collins, Colorado

**NHDP = National Hansen’s Disease Program

### Basic characteristics of leprosy patients and controls with other confounding dermatoses

One hundred forty-five leprosy cases (n = 145) [MB (before MDT), n = 70; PB (before MDT), n = 46; and MB (post MDT), n = 14; PB (post MDT), n = 15] and 29 controls [piebald, n = 5; psoriasis, n = 5; pityriasis rosea, n = 5; body moss, n = 5; multiple neurofibroma, n = 2; multiplex lipoid granuloma, n = 2; and scleroderma, n = 5] from the same endemic region were included. The basic information for each study group is summarized in Table 3.

**Table 3:** Clinical characteristics of FFPE specimens of the leprosy patients enrolled in this study.

Leprosy	Leprosy classification (n, %)			Cases (n)	Sex ratio (M/F)	Year (range)	Bacterial index (BI)
	WHO*	RJ**					
Before MDT	MB	LL	8	70	6/2	15–64	BI = 5, n = 4; BI = 6, n = 4.
		BL	37		22/15	17–79	BI = 1, n = 2; BI = 2, n = 4; BI = 3, n = 5; BI = 4, n = 21; BI = 5, n = 4.
		BB	1		1/0	43	BI = 1, n = 1
		BT	24		16/8	10–73	BI = 1, n = 18; BI = 2, n = 4; BI = 3, n = 2.
	PB	BT	31	46	14/17	12–76	BI = 0
		TT	15		9/6	9–72	BI = 0
After MDT	MB	LL	9	14	8/1	14–67	BI = 0, n = 3; BI = 1, n = 3; BI = 2, n = 3.
		BL	2		1/1	79–79	BI = 0.8, n = 1; BI = 0, n = 1
		BB	3		2/1	18–35	BI = 0
	PB	BT	7	15	5/2	19–64	BI = 0
		TT	8		6/2	12–77	BI = 0

n: number of patients, with percentages in parentheses.

*WHO: Operational classification proposed by the World Health Organization.

** RJ: Ridley-Jopling classification.

### Clinical evaluation of sensitivity, specificity, and accuracy of tests

Of the 174 specimens included in the present study, 145 patients were diagnosed as having leprosy based on clinical signs and symptoms, skin smears, skin biopsy, and neurophysiologic examinations according to the leprosy diagnosis criteria of China. Other confounding dermatoses were diagnosed based on 29 specimens. Of the 145 leprosy patients, 116 cases were before MDT (MB, n = 70; PB, n = 46) and 29 after MDT (MB, n = 14, PB, n = 15). Using these criteria, the sensitivity, specificity, PPV, NPV, LRs and accuracy of the individual methods were calculated.

The sensitivities of the BI, simple PCR, STNPCR, and SYBRGreen PCR were 60.34%, 42.24%, 67.92%, and 60.34% for leprosy patients, respectively (Table 3). All of these tests had a specificity of 100%. The PPVs of the BI, simple PCR, STNPCR, and SYBRGreen PCR assays were all 100%, and the NPVs were 38.66%, 30.20%, 40.03%, and 38.66%, respectively. Positive and negative LRs and accuracy were also calculated. For leprosy patients, STNPCR showed the highest sensitivity, specificity, PPV and NPV (Table 4). STNPCR also demonstrated the highest positive LR of infinity and the highest accuracy of 74.81% but the lowest negative LR of 32.07% (Table 4).

**Table 4:** Comparison of sensitivity, specificity, positive predictive value (PPV), negative predictive value (NPV), likelihood ratio (LRs) and accuracy of different methods calculated with 95% confidence intervals (CIs) for FFPE specimens from MB, PB and leprosy patients before MDT.

Result for specimen	BI		Simple PCR		STNPCR		SYBRGreen	
(n = 99)	Positive	Negative	Positive	Negative	Positive	Negative	Positive	Negative
MB (before MDT) Positive	70	0	41	29	64	6	57	13
Leprosy Negative	0	29	0	29	0	29	0	29
Sensitivity (95% CI)	100(93.51–100)		58.57(46.17–70.01)		91.42(81.64–96.46)		81.42(69.97–89.36)	
Specificity (95% CI)	100(85.43–100)		100(85.43–100)		100(85.43–100)		100(85.43–100)	
PPV (95% CI)	100(93.51–100)		100(89.33–100)		100(92.94–100)		100(92.13–100)	
NPV (95% CI)	100(85.43–100)		50.00(36.73–63.26)		82.85(65.70–92.83)		69.04(52.75–81.88)	
Positive Likelihood Ratio (LR+)	Infinity(NaN-Infinity)		Infinity(NaN-Infinity)		Infinity(NaN-Infinity)		Infinity(NaN-Infinity)	
Negative Likelihood Ratio (LR-)	0(0-NaN)		41.42(31.35–54.73)		8.57(3.98–18.42)		18.57(11.37–30.33)	
Accuracy %	100.00		70.70		93.93		86.86	
Result for specimen	BI		Simple PCR		STNPCR		SYBRGreen	
(n = 75)	Positive	Negative	Positive	Negative	Positive	Negative	Positive	Negative
PB (before MDT) Positive	0	46	8	38	18	28	13	33
Leprosy Negative	0	29	0	29	0	29	0	29
Sensitivity (95% CI)	0(0–0.0.09)		17.39(8.32–31.95)		39.13(25.45–54.60)		28.26(16.45–43.68)	
Specificity (95% CI)	100(85.43–100)		100(85.43–100)		100(85.43–100)		100(85.43–100)	
PPV (95% CI)	NaN(NaN-NaN)		100(59.77–100)		100(78.12–100)		100(71.65–100)	
NPV (95% CI)	38.66(27.85–50.64)		43.28(31.42–55.91)		50.87(37.43–64.20)		46.77(34.16–59.78)	
Positive Likelihood Ratio (LR+)	NaN(NaN-NaN)		Infinity(NaN-Infinity)		Infinity(NaN-Infinity)		Infinity(NaN-Infinity)	
Negative Likelihood Ratio (LR-)	1(1–1)		82.60(72.35–94.32)		60.86(48.28–76.74)		71.73(59.83–86.00)	
Accuracy %	38.67		49.33		62.67		56.00	
Result for specimen	BI		Simple PCR		STNPCR		SYBRGreen	
(n = 145)	Positive	Negative	Positive	Negative	Positive	Negative	Positive	Negative
Leprosy (before MDT) Positive	70	46	49	67	72	34	70	46
Leprosy Negative	0	29	0	29	0	29	0	29
Sensitivity (95% CI)	60.34(50.81–69.17)		42.24(33.23–51.76)		67.92(58.05–76.47)		60.34(50.81–69.17)	
Specificity (95% CI)	100(85.43–100)		100(85.43–100)		100(85.43–100)		100(85.43–100)	
PPV (95% CI)	100(93.51–100)		100(90.94–100)		100(93.68–100)		100(93.51–100)	
NPV (95% CI)	38.66(27.85–50.64)		30.20(21.47–40.55)		46.03(33.57–58.97)		38.66(27.85–50.64)	
Positive Likelihood Ratio (LR+)	Infinity (NaN-Infinity)		Infinity (NaN-Infinity)		Infinity (NaN-Infinity)		Infinity (NaN-Infinity)	
negative Likelihood Ratio (LR-)	39.65(31.68–49.63)		57.75(49.43–67.48)		32.07(24.31–42.31)		39.65(31.68–49.63)	
Accuracy %	68.27		53.79		74.81		68.27	

For MB patients before MDT, STNPCR showed the highest sensitivity of 91.42%, the highest specificity of 100%, a PPV of 100%, an NPV of 82.85%, and the highest accuracy of 93.93%, which were higher than those for simple PCR but lower than those for BI.

For PB patients before MDT, STNPCR showed the highest sensitivity of 39.13%, the highest specificity of 100%, a PPV of 100%, an NPV of 50.87%, and the highest accuracy of 62.67%, which were higher than those of SYBRGreen PCR, simple PCR, and BI.

### Monitoring response to MDT by RLEP PCR

Twenty-nine leprosy patients in this study were under MDT at the CDC and had finished MDT when enrolled in the study. The BI, simple PCR, STNPCR, and SYBRGreen PCR assays using RLEP primers were performed with specimens obtained from these patients after MDT to determine the presence of target DNA to monitor the response to MDT. There were 22 patient specimens in which the BI was zero, whereas the remaining 76 patient specimens had a positive BI. Among the 22 patient specimens (BI = 0), RLEP PCR revealed 3, 6, and 5 positive amplifications of patient specimens by simple PCR, STNPCR, and SYBRGreen PCR, respectively. Among the 6 patient specimens (BI = positive), RLEP PCR resulted in 2, 4, and 5 positive amplifications of patient specimens by simple PCR, STNPCR, and SYBRGreen PCR, respectively (Table 5).

**Table 5:** Comparison between PCR assays and BI/techniques using FFPE specimens after MDT.

FFPE specimens of post-MDT	Simple PCR Positive (n, %)	STNPCR Positive (n, %)	SYBRGreen PCR Positive (n, %)
BI negative (n = 22)	3 (13.63%)	6 (27.27%)	5 (22.72%)
BI positive (n = 7)	2 (28.57%)	4 (57.14%)	5 (71.42%)

Compared with STNPCR and simple PCR, SYBRGreen PCR exhibited the highest sensitivities of 50.00% for MB and 34.48% for leprosy patients and the highest accuracies of 83.72% for MB and 67.24% for leprosy patients. However, for PB patients, STNPCR showed the highest sensitivity (26.88%) and the highest accuracy (75.00%) compared with SYBRGreen PCR and simple PCR. (Table 6).

**Table 6:** Comparison of sensitivity, specificity, positive predictive value (PPV), negative predictive value (NPV), likelihood ratio (LRs) and accuracy of different methods calculated with 95% confidence intervals (CIs) for MB, PB, and leprosy patients after MDT.

Result for specimen	BI		Simple PCR		STNPCR		SYBRGreen	
(n = 43)	Positive	Negative	Positive	Negative	Positive	Negative	Positive	Negative
MB (after MDT) Positive	7	7	3	11	6	8	7	7
MB (after MDT) Negative	0	29	0	29	0	29	0	29
Sensitivity (95% CI)	50(24.04–75.95)		21.42(5.70–51.1)		42.85(18.81–70.35)		50.00(24.04–75.95)	
Specificity (95% CI)	100(85.43–100)		100(85.43–100)		100(85.43–100)		100(85.43–100)	
PPV (95% CI)	100(56.09–100)		100(30.99–100)		100(51.68–100)		100(56.09–100)	
NPV (95% CI)	80.55(63.43–91.19)		72.50(55.86–84.85)		78.37(61.33–89.57)		80.55(63.43–91.1)	
Positive Likelihood Ratio (LR+)	Infinity(NaN-Infinity)		Infinity(NaN-Infinity)		Infinity(NaN-Infinity)		Infinity(NaN-Infinity)	
Negative Likelihood Ratio (LR-)	50.00(29.61–84.42)		78.57(59.76–103.29)		57.14(36.30–89.94)		50.0(29.61–84.42)	
Accuracy %	83.72		74.41		81.39		83.72	
Result for specimen	BI		Simple PCR		STNPCR		SYBRGreen	
(n = 44)	Positive	Negative	Positive	Negative	Positive	Negative	Positive	Negative
PB (after MDT) Positive	0	15	2	13	4	11	3	12
PB (after MDT) Negative	0	29	0	29	0	29	0	29
Sensitivity (95% CI)	0(0–25.34)		13.33(2.34–41.61)		26.66(8.91–55.16)		20.00(5.31–48.62)	
Specificity (95% CI)	100(85.43–100)		100(85.43–100)		100(85.43–100)		100(85.43–100)	
PPV (95% CI)	NaN(NaN-NaN)		100(19.78–100)		100(39.57–100)		100(30.99–100)	
NPV (95% CI)	65.90(49.99–79.06)		69.04(52.75–81.88)		72.50(55.86–84.85)		70.73(54.26–83.35)	
Positive Likelihood Ratio (LR+)	NaN(NaN-NaN)		Infinity(NaN-Infinity)		Infinity(NaN-Infinity)		Infinity(NaN-Infinity)	
Negative Likelihood Ratio (LR-)	100(100–100)		86.66(71.06–105.69)		73.33(54.04–99.50)		80.00(62.11–103.03)	
Accuracy %	65.90		70.45		75.00		72.72	
Result for specimen	BI		Simple PCR		STNPCR		SYBRGreen	
(n = 56)	Positive	Negative	Positive	Negative	Positive	Negative	Positive	Negative
Leprosy (after MDT) Positive	7	22	5	24	10	19	10	19
Leprosy (after MDT) Negative	0	29	0	29	0	29	0	29
Sensitivity (95% CI)	24.13(11.01–43.92)		17.24(6.52–36.48)		34.48(18.59–54.33)		34.48(18.59–54.33)	
Specificity (95% CI)	100(85.43–100)		100(85.43–100)		100(85.43–100)		100(85.43–100)	
PPV (95% CI)	100(56.09–100)		100(46.29–100)		100(65.54–100)		100(65.54–100)	
NPV (95% CI)	50.00(36.73–63.26)		54.71(40.55–68.19)		60.41(45.29–73.88)		60.41(45.29–73.88)	
Positive Likelihood Ratio (LR+)	56.86(42.32–70.37)		Infinity(NaN-Infinity)		Infinity(NaN-Infinity)		Infinity(NaN-Infinity)	
Negative Likelihood Ratio (LR-)	100(100–100)		82.75(70.09–97.71)		65.51(50.31–85.31)		65.51(50.31–85.31)	
Accuracy %	62.06		58.62		67.24		67.24	

## Discussion

Early diagnosis of the disease, followed by prompt treatment with MDT, is the basis of current leprosy control strategies [16]. To prevent continued transmission and misdiagnosis, better tests and more efficient diagnostic procedures are needed in hyperendemic areas of leprosy in developing countries.

More than 100 counties, mainly in southwestern China, report incidence rates of leprosy >1/100,000 [17]. FFPE, which has the advantage of being used for remote transfer or retrieval, is the most common specimen that can be attained by clinical molecular laboratories. This study was designed to develop and assess the performance of new primer sets (131 bp and 101 bp) versus published primers (372 bp) for RLEP in PCR-based diagnosis of *M*. *leprae* and to evaluate the diagnostic performance and monitor the developed STNPCR and SYBRGreen PCR assays using primers specific for the RLEP gene in FFPE specimens.

Culturing *M*. *leprae* is very difficult, and traditional laboratory techniques, such as the BI, are far from sensitive and specific. In the absence of a true gold standard, PCR methods, such as quantitative PCR (qPCR), multiplex PCR, and droplet digital PCR assays, have been developed to aid the diagnosis of leprosy worldwide [18–23].

Because acid-fast staining requires at least 10^4^ organisms per gram of tissue for reliable detection, the sensitivity of this method is low, particularly for patients at the TT end of the leprosy spectrum in which AFBs are rare or absent. The specificity of this approach is also subject to error primarily because *Mycobacteria* other than *M*. *leprae* will also stain positive. Therefore, the development of a rapid, simple, and specific detection of small numbers of *M*. *leprae* in tissue samples would considerably help in the diagnosis of leprosy and possibly lead to better strategies for more rapid initiation of treatment [24]. In general, detection of *M*. *leprae*-specific DNA sequences might represent a more sensitive and quicker diagnostic approach. The species specificity of the two primer sets was also examined in this study.

The PCR methods with two primer sets were less accurate than BI for leprosy diagnosis among MB patients [STNPCR, SYBRGreen, simple PCR, and BI: 93.93%, 86.86%, 70.70% and 100% for MB, respectively]. However, STNPCR with the primer set amplifying the 101-bp fragment used in the present study for FFPE samples showed reasonable accuracy, whereas simple PCR methods for FFPE and BI for skin slit smear were less accurate [STNPCR, SYBRGreen, simple PCR, and BI: 62.67%, 56.00%, 49.33% vs 38.67% for PB, respectively, and 74.81%, 68.27%, 53.79% vs 68.27% for leprosy (before MDT), respectively]. RLEP STNPCR was the most accurate assay compared to simple PCR and BI for PB and leprosy diagnoses.

For monitoring response to MDT, the SYBRGreen PCR with the 101-bp fragment primer set used in the present study for FFPE showed reasonable accuracy, but the simple PCR methods for FFPE were less accurate [STNPCR, SYBRGreen, simple PCR, and BI: 81.39%, 83.72%, 74.41% vs 83.72% for MB, respectively; 75.00%, 72.72%, 70.45% vs 65.90% for PB; and 67.27%, 67.27%, 58.62%, and 62.06% for leprosy (after MDT), respectively]. The RLEP SYBRGreen PCR was the most accurate assay for monitoring MDT response compared to the other methods for MB, whereas STNPCR had the greatest accuracy for monitoring MDT response in PB and leprosy patients after treatment. Both STNPCR and SYBRGreen PCR (101-bp fragment) showed better accuracy than simple PCR (372-bp fragment) in leprosy diagnosis and in MDT monitoring.

Conventional nested PCR is a very sensitive and specific method for the diagnosis of pathogens. However, this type of PCR is notorious for contamination problems related to the processing of the product between the first and second PCR steps [25]. In the present study, we found an extremely high positivity rate for the diagnosis of *M*. *leprae* by conventional nested PCR using RLEP (101-bp fragment) as the target. To obtain a PCR method that is just as efficient but without the risk of contamination, we optimized STNPCR. In the case of STNPCR and SYBRGreen PCR, the internal primers (101 bp) were developed within the published primer (372 bp) region of *M*. *leprae* RLEP. We also used UNG in STNPCR, and the UNG-treated loop-mediated isothermal amplification (LAMP) assay can prevent unwanted amplification by carryover contamination of the previously amplified DNA [26]. The STNPCR and SYBRGreen PCR assays were optimized to minimize the duration of the PCR protocol (2 h and 1 h, respectively) and can detect even low levels of template (13 fg) genomic DNA.

The sensitivity of RLEP PCR is reported to be 57%-80% for the diagnosis of leprosy. When evaluated using clinical specimens, AFB positivity in skin biopsy was 44% (range 10–85), whereas PCR positivity in skin biopsy was 70% (range 46–93) [27]. When comparing different PCR methods, the highest percentage of PCR sensitivity was observed using the multiplex PCR technique (82%), followed by RT-PCR (78%) and conventional PCR (63%) [27]. We observed a sensitivity of 58.57%-91.42% for FFPE from MB patients and 17.39%-39.13% for FFPE from PB patients but better specificity (100%) with the STNPCR and SYBRGreen primers. Because skin biopsy is the most frequent clinical procedure used to collect samples for leprosy diagnosis and RLEP is the most frequent marker used, suitable tools, such as RLEP PCR for FFPE specimens, for providing bacteriological evidence of leprosy are needed for early case detection and appropriate therapeutic management [27].

In addition to the *M*. *leprae* diagnostic studies, PCR assays using two primer sets for *M*. *leprae* RLEP were carried out to monitor the response to anti-*M*. *leprae* MDT. Therefore, FFPE specimens were collected from leprosy patients after MDT, from both BI-positive and BI-negative patients given MDT. The most common methods for monitoring the response to MDT are a progressive reduction in the number of AFBs in skin slit smears and histological examination of skin lesion biopsies. The present method using RLEP PCR can be employed to semi-quantitate *M*. *leprae* DNA to assess a patient’s response to MDT.

Our study has limitations. We tested our quantitative PCR assay for *M*. *leprae* RLEP for the diagnosis of PB leprosy patients from a hyperendemic area of China [14,28]. More rigorous studies from low-prevalence regions are needed to better define and confirm the diagnostic applicability of this assay. In addition, our study involved a small cohort, especially for PB leprosy, and there were differences in the before and after MDT subgroups. Furthermore, the methodology did not include current PCR methods, with the best results being multiplex PCR as well as RT-PCR.

In conclusion, we developed an assay to amplify 131-bp and 101-bp fragments within the published 372-bp fragment of RLEP to identify *M*. *leprae* DNA in FFPE specimens. Moreover, we used UNG in the STNPCR assay to reduce the number of steps and prevent contamination when compared with conventional nested PCR. The detection limitation for the purified *M*. *leprae* DNA was 13 fg using simple PCR, STNPCR and SYBRGreen PCR. We also assessed the specificity of the system against other *Mycobacterium* and non-*Mycobacterium* species. Within the context of clinical evolution, amplification of the 131-bp and 101-bp fragments of RLEP in FFEP specimens from leprosy patients performs better as a diagnostic test and for monitoring response to MDT than simple PCR based on amplification of the 372-bp fragment. Despite being regarded as suboptimal material for molecular diagnosis, given their use in routine tests for suspected leprosy and their ability to be transferred remotely or retrieved from previous patients, FFPE specimens are still essential material for *M*. *leprae* identification.
